# A surgical case of high-grade urothelial carcinoma of the renal pelvis complicated with giant hydronephrosis, giving rise to diagnostic difficulties on a cytological examination

**DOI:** 10.1186/s13000-022-01227-2

**Published:** 2022-05-11

**Authors:** Mao Takata, Motona Kumagai, Yumi Tsubata, Yoshiiku Okanemasa, Michiho Takenaka, Toshie Terauchi, Manabu Yamashita, Akihiro Shioya, Sohsuke Yamada

**Affiliations:** 1 Kanazawa Medical University Hospital, 1-1, Ishikawa 920-0293 Uchinada, Japan; 2 Department of Pathology and Laboratory Medicine, Kanazawa Medical University, Uchinada, Japan; 3 Department of Pathology II, Kanazawa Medical University, Uchinada, Japan

**Keywords:** Renal pelvis, Urothelial carcinoma, Hydronephrosis, Cytology, Squamous differentiation

## Abstract

**Background:**

We report a surgical case of urothelial carcinoma of the renal pelvis, resulting in diagnostic difficulties on cytological examination.

**Case presentation:**

A man in his late 70s underwent nephrectomy for giant hydronephrosis and renal cysts after nephrostomy and renal cyst puncture and drainage. On all cytological examinations performed before surgery, including nephrostomy urine, renal cyst fluid, catheterized bladder urine, and bladder washings, we were unable to make any conclusive diagnosis of malignancy. The pathological diagnosis of the surgical specimen concluded that this was a case of high-grade urothelial carcinoma of the renal pelvis with focal squamous differentiation (pT4). Liver and lung metastases were identified 3 months after surgery, and the patient died 2 months later.

**Conclusion:**

It was very difficult to make a conclusive diagnosis using cytological specimens because of the presence of a small number of atypical cells with severe degenerative changes. Since clinicians cannot predict the potential for malignancy on preoperative imaging findings, it is critical to consider the difficulties in clinically making a correct diagnosis of urothelial carcinoma of the upper urinary tract, especially in cases complicated with giant hydronephrosis.

## Background

Urothelial carcinoma of the renal pelvis is a malignant tumor that accounts for approximately 5% of all urothelial tumors, and is rarer than bladder cancer. It is more common in males than females and occurs between 50 and 70 years of age [1]. Over 60% of cases are already invasive at the time of diagnosis, and it is regarded as a tumor with a poor prognosis [2]. It is diagnosed using a combination of computed tomography (CT) urography, retrograde pyeloureterography, urine cytology and/or biopsy, prior to nephroureterectomy. The sensitivity of spontaneous urine cytology is lower than that of bladder cancer and selective cytology should be performed in the upper urinary tract [3, 4]. Histologically, > 90% of carcinomas of the urinary system are urothelial carcinomas, squamous cell carcinoma, adenocarcinoma, small cell carcinoma, and undifferentiated carcinoma also occur rarely [5]. Urothelial carcinoma with squamous differentiation is the most frequent type and is reported to be found in approximately 16% of upper urothelial carcinomas [6]. In the present study, we report a case of urothelial carcinoma of the renal pelvis associated with giant hydronephrosis that was difficult to diagnose despite multiple cytological examinations.

## Case presentation

**Fig. 1:**
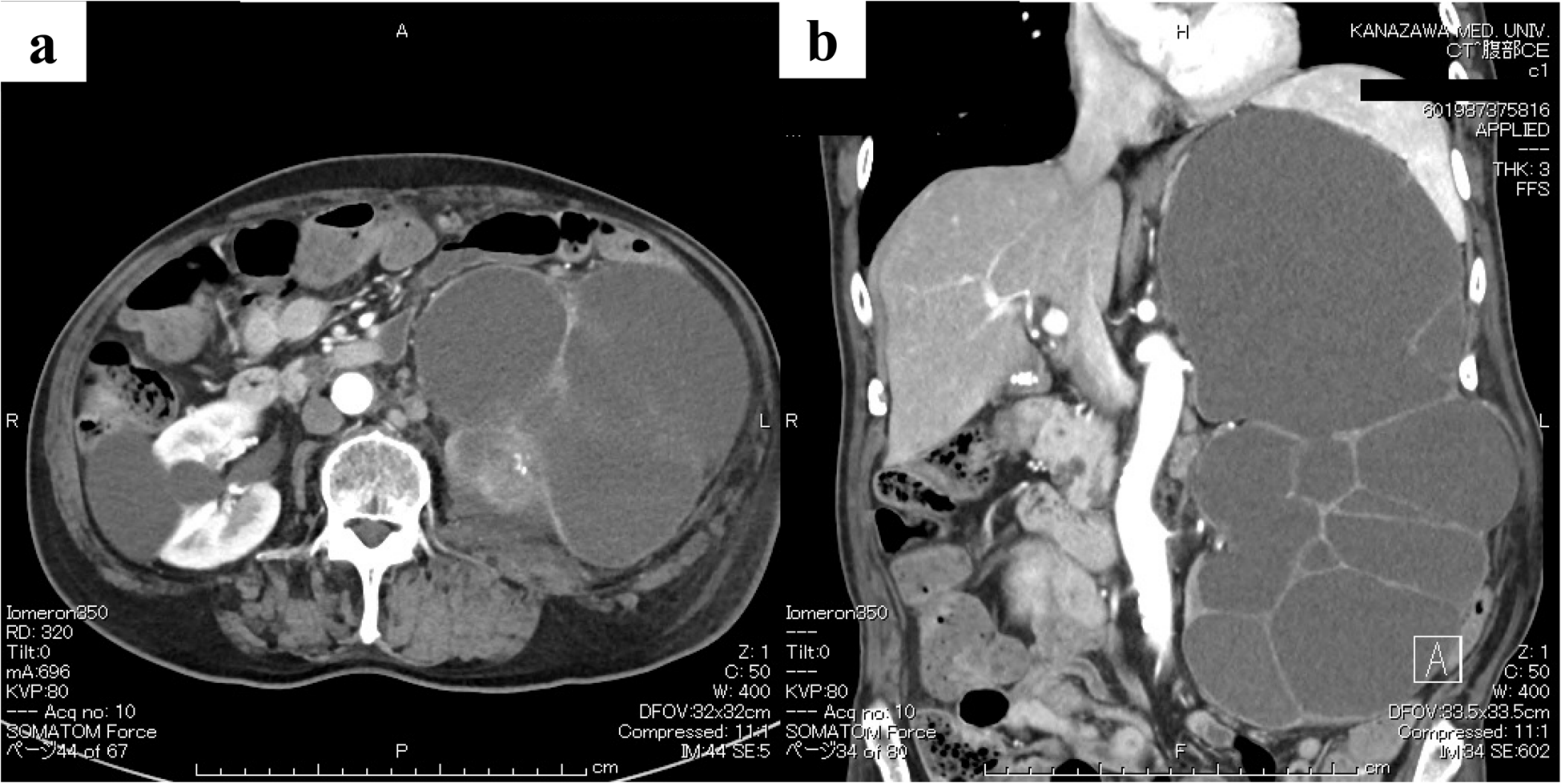
Computed tomography of the abdomen before nephrostomy. The left kidney was multicystic and markedly enlarged

A man in his late 70s (76-year-old) had a history of left kidney stones, hypertension, and early-stage gastric cancer. He had been diagnosed with left hydronephrosis and left and right renal cysts 15 years ago; however, his urine occult blood test results were negative, and he was under observation. In September XXXX, he visited the Department of Hepatology, Biliary and Pancreatic Diseases because he was aware of a bulge and pain in his left abdomen. At that time, he was referred to the Department of Urology because ultrasonography showed marked exacerbation of the left hydronephrosis. CT showed marked enlargement of the left kidney (Fig. 1) and bladder dilatation. He underwent urinary catheterization for suspected aggravation of hydronephrosis due to urinary retention; however, the hydronephrosis did not improve. Nephrostomy, cystocentesis, and drainage were performed in October. The patient showed improvement, but in January XXX1, fever and hematuria developed. Imaging studies revealed suspected hemorrhage in the left renal cyst. Although there was no clinical evidence of malignancy, the left kidney was in a non-functional state, and nephrectomy was performed in March. During the period between urological consultation and surgery, cytological examination was performed with four types of materials: nephrostomy urine, renal cystocentesis fluid, indwelling catheter urine, and bladder lavage fluid. However, urothelial carcinoma could not be diagnosed preoperatively. The patient was pathologically diagnosed with urothelial carcinoma (pT4) arising from the renal pelvis [5]. Three months after the surgery, liver and lung metastases appeared, and chemotherapy was administered. The patient died 5 months after surgery.

For renal cyst puncture fluid, specimens were centrifuged at 1500 rpm for 5 min, and the sediment was smeared using the grating method. For nephrostomy urine, catheter urine, and bladder washing, specimens were centrifuged at 1500 rpm for 5 min, and 2% polyethylene glycol and 70% ethanol were added to the sediment, followed by cell collection and smearing using an autosmear.

### Nephrostomy

**Fig. 2:**
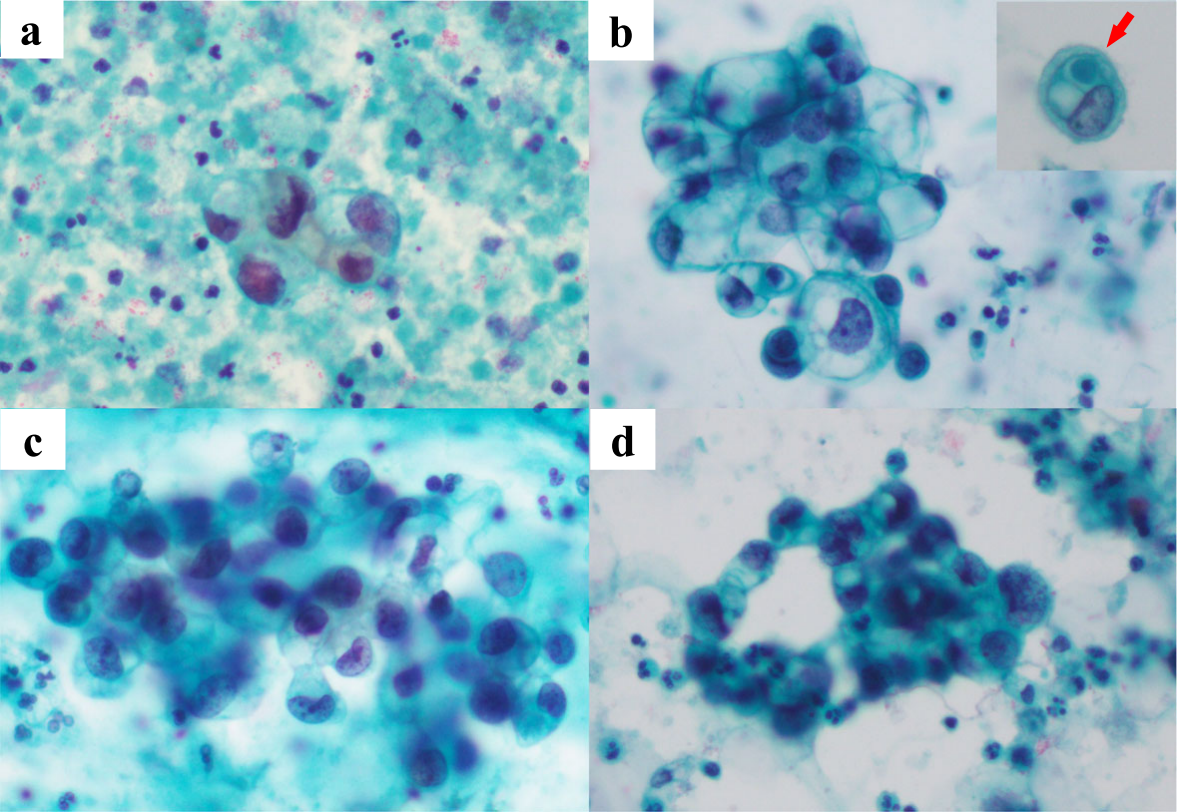
Cytological findings. A small number of degenerative atypical cells with enlarged and irregular nuclei in the hemorrhagic and inflamed background (**a**: left nephrostomy urine). Atypical cell clusters with focal clear cytoplasm. The inset indicates a representative type A intracytoplasmic lumen with a secretory globule (arrow) (**b**: left renal cyst fluid). Slightly large clusters of degenerative atypical cells (**c**: catheterized bladder urine). Overtly atypical cell clusters with high N/C ratios (**d**: bladder washings) (All photos; Papanicolaou staining, × 40)

Atypical cells with enlarged nuclei and irregular nuclear shapes were observed in the inflammatory background with hemorrhage (Fig. 2a). Because of the small number of atypical cells and high degree of cellular degeneration, it was difficult to differentiate between benign and malignant cells. Finally, the cells were judged to be atypical.

### Renal cyst puncture fluid

Among the large amounts of necrotic material, inflammatory cells, and red blood cells, atypical cells with enlarged nuclei and uneven nuclear size appeared in small clusters (Fig. 2b). The presence of vacuolated, pale cytoplasm led to a diagnosis of clear cell renal cell carcinoma. An additional smear was prepared, and periodic acid-Schiff (PAS) staining was performed. The cytoplasm was negative for PAS staining, which is unusual for clear cell renal cell carcinoma. Immunostaining using the cell transcription method showed that atypical cells were positive for GATA3 and negative for PAX8, indicating that the cells were not renal carcinoma. Cell atypia was too mild for suspicion of high-grade urothelial carcinoma, and the cells were judged to be atypical. In addition, intracytoplasmic lumina (ICLs) were observed in some atypical cells.

### Catheterized bladder urine

On the hemorrhagic background, there were slightly large clusters of degenerative atypical cells (Fig. 2c), similar to those in the renal cyst puncture fluid, as described above. These atypical cells showed enlarged and irregular-shaped nuclei with nuclear maldistribution. Since the possibilities of those degenerative change could not be excluded out, the mildly hyperchromatic cells were cytologically diagnosed merely as atypical.

### Cystourethral lavage fluid

On the hemorrhagic background, some atypical cells with a slightly higher nuclear-cytoplasmic (N/C) ratio than the previous test results were observed (Fig. 2d). These atypical cells were suspected to be high-grade urothelial carcinoma, as they showed increased nuclear chromatin, irregular-shaped nuclei, high N/C ratio, nuclear maldistribution, and nuclear enlargement. Because only a small number of cells appeared, it was judged as suspicious for malignancy.

Since ICLs were found in the renal cyst puncture fluid, we focused on the ICLs and performed specimen recirculation. ICLs are classified as type A, with secretions in the lumen, or type B, without secretions [7]. The number and type of ICLs appearing were measured in nephrostomy urine, renal cystocentesis fluid, indwelling catheter urine, and bladder lavage fluid. The results of recanalization showed no ICLs in nephrostomy urine, but they were found in specimens of renal cyst perforation fluid and urine from indwelling bladder catheters (Table 1).

**Table 1:** Intracytoplasmic lumina in cytology specimen

Specimen	Number of ICLs	
	Type A	Type B
Left nephrostomy urine	0	0
Left renal cyst fluid	5	1
Catheterized bladder urine	4	3
Bladder washings	3	2

*ICL* Intracytoplasmic lumina

**Fig. 3:**
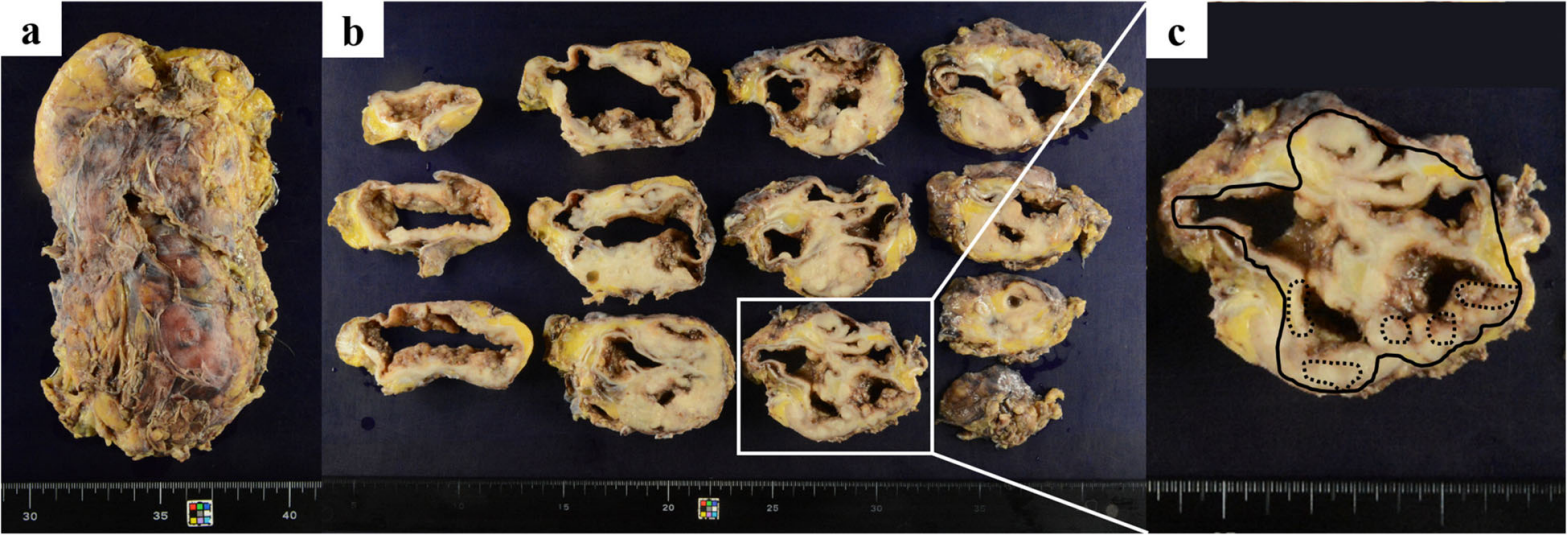
Macroscopic findings in the resected specimen of a markedly enlarged left kidney (**a**). The cut surface appeared multicystic and solid, and most of the renal parenchyma was replaced with a whitish mass (**b**). The solid line shows high-grade urothelial carcinoma components, whereas several parts of the squamous cell differentiation components are indicated by dashed lines (**c**)

**Fig. 4:**
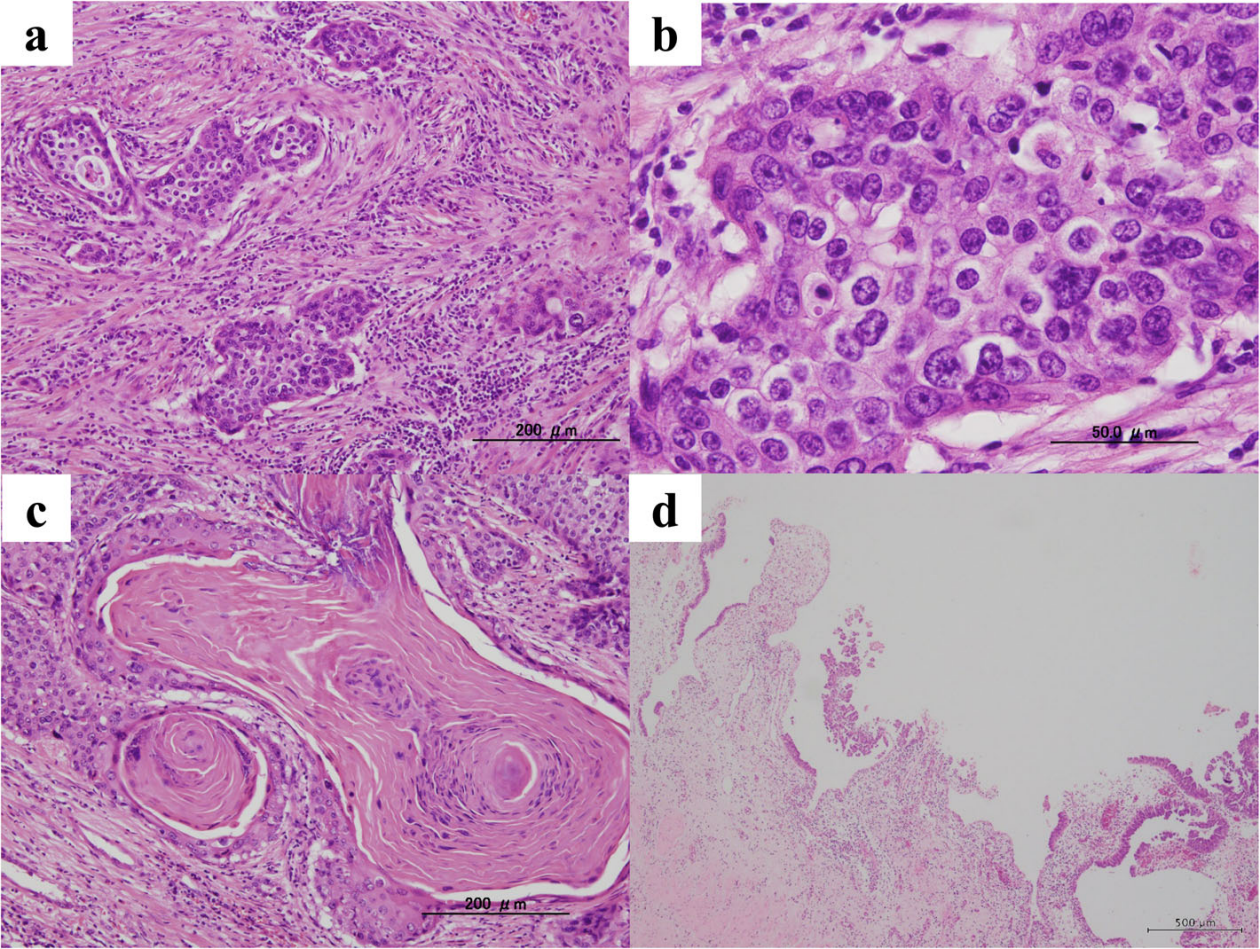
Histological findings in the surgical specimen. The tumor shows an invasive growth pattern (**a**; scale bar = 200 μm, hematoxylin and eosin [H&E] staining; × 10) and solid/diffuse proliferation of highly atypical urothelial-like cells with large, irregular, and pleomorphic nuclei (**b**; scale bar = 50 μm, H&E staining, × 40). Apparent keratinization was only partly observed. **c**; scale bar = 200 μm, H&E staining, × 10). Non-neoplastic uroepithelium (left) and adjacent focus of urothelial carcinoma in situ (right) are present in the renal pelvis. **d**; scale bar = 500 μm, H&E staining, × 4)

The excised left kidney was 165 × 90 mm, 339 g, and the excised material consisted almost entirely of masses (Fig. 3a). On the circumferential surface, a substantial cystic whitish mass was observed, and most of the renal parenchyma was replaced by these neoplastic lesions (Fig. 3b). Both the renal pelvis and ureter were dilated, and no obvious stenosis was noted. Histologically, the tumor showed diffuse growth of spindle-shaped, round to polygonal, highly atypical cells with large irregular nuclei in a plump to irregular pattern (Fig. 4a, b). Some of the lesions were clearly keratinized and differentiated into squamous epithelium (Fig. 4c). Intraepithelial carcinoma was also observed in the renal pelvis. The non-neoplastic pyeloureteral epithelium and intraepithelial carcinoma were near each other, and although direct continuity was not apparent owing to epithelial shedding, it was thought that there was continuity (Fig. 4d). The intraepithelial component of the tumor extended into the ureter. The tumor invaded the perirenal adipose tissue from the renal pelvis across the renal parenchyma and left adrenal gland, which was resected at the same time. The left ureteral margin was negative. Immunohistochemically, the tumor cells were positive for CK7 and p63, partially positive for CK20, negative for PAX8 and vimentin, and were diagnosed as high-grade urothelial carcinoma (pT4) with squamous differentiation [5]. The distributions of high-grade urothelial carcinoma and squamous cell carcinoma are shown in Fig. 3c.

## Discussion & conclusion

In this case, the cytological specimen was re-examined after the diagnosis of postoperative urothelial carcinoma was confirmed. In the initial nephrostomy urine sample, multiple findings were suggestive of high-grade urothelial carcinoma. However, since the appearance of atypical cells was very small and degeneration was severe, we judged the case as suspicious for malignancy. In renal cyst puncture fluid, degenerative findings such as nuclear enrichment and intracytoplasmic vacuoles were observed in many cells. Furthermore, because there were no well-preserved benign urothelial cells, we could not compare nuclear chromatin enrichment (or nuclear enrichment) with benign cells, which is considered a critical factor in the diagnosis of high-grade uroepithelial carcinoma. The low N/C ratio of atypical cells in nephrostomy urine, renal cyst perforation fluid, and urine from indwelling catheters was one of the factors that made us hesitate to suspect a malignancy. In the cystoureteral lavage fluid, atypical weak cells similar to those seen in the renal cyst puncture fluid appeared; however, some atypical cells with gold-standard findings suggestive of high-grade urothelial carcinoma appeared. Although the number of atypical cells was small, preservation of the cells was relatively good, and the specimen was considered malignant. The squamous cell differentiation component observed in the histological specimen did not appear in the cytological specimen.

In addition to the gold-standard diagnostic criteria for high-grade urothelial carcinoma, we focused on ICLs, which are an important cytological indicator in breast cancer. Terauchi et al. [7] reported that the incidence of type A ICL in urine cytology specimens was significantly higher in highly heterozygous urothelial carcinoma than in homozygous urothelial carcinoma and was not observed in non-neoplastic cases. They also stated that type A ICL may contribute to the diagnosis of urothelial carcinoma. In the study by Terauchi et al., ICL was considered positive when two or more type A ICLs were found in a specimen. In the case we report here, renal cyst perforation fluid, urine from an indwelling bladder catheter, and bladder lavage fluid showed more than three type A ICLs per specimen. This finding suggests a high-grade urothelial carcinoma. In this case, in addition to the small number of atypical cells and high degree of cellular degeneration, it was difficult to determine whether there was an increase in nuclear chromatin (or dark staining). Therefore, it was difficult to make decisions. It is not sufficient to simply comment that it is difficult to differentiate between benign and malignant cells in specimens reported as atypical. We should emphasize that the presence of high-grade urothelial carcinoma cannot be ruled out. Kuromoto et al. [8] reported a case of difficult preoperative diagnosis of renal pelvis cancer complicated by megahydronephrosis. In their report, they noted that the positive rate of pyelography in renal pelvis carcinoma complicated by megahydronephrosis was as low as 4 out of 10 cases (40%) and that malignancy should be considered when the renal pelvis solution is bloody. For the preoperative diagnosis and selection of the operative procedure, it is important to know with the urologist that the cytological diagnosis of pyelocarcinoma associated with giant hydronephrosis is difficult.

Here, we report a case of urothelial carcinoma of the renal pelvis associated with massive hydronephrosis. Diagnosis was difficult because of the small number of atypical cells and cell degeneration. It is important to note that upper urinary tract epithelial carcinoma associated with giant hydronephrosis is difficult to diagnose preoperatively.

## Data Availability

The dataset supporting the findings and conclusions of this case report is included within the article.

## References

[1] Hall MC, Womack S, Sagalowsky AI, Carmody T, Erickstad MD, Roehrborn CG (1998). Prognostic factors, recurrence, and survival in transitional cell carcinoma of the upper urinary tract: a 30-year experience in 252 patients. Urology..

[2] Shao IH, Chang YH, Pang ST (2019). Recent advances in upper tract urothelial carcinomas: from bench to clinics. Int J Urol.

[3] Messer J, Shariat SF, Brien JC, Herman MP, Ng CK, Scherr DS, Scoll B, Uzzo RG, Wille M, Eggener SE, Steinberg G, Terrell JD, Lucas SM, Lotan Y, Boorjian SA, Raman JD (2011). Urinary cytology has a poor performance for predicting invasive or high-grade upper-tract urothelial carcinoma. BJU Int.

[4] Rouprêt M, Babjuk M, Burger M, Capoun O, Cohen D, Compérat EM, Cowan NC, Dominguez-Escrig JL, Gontero P, Hugh Mostafid A, Palou J, Peyronnet B, Seisen T, Soukup V, Sylvester RJ, Rhijn BWG, Zigeuner R, Shariat SF (2021). European Association of Urology guidelines on upper urinary tract urothelial carcinoma: 2020 update. Eur Urol.

[5] Moch H, Humphrey PA, Ulbright TM, Reuter V (2016). WHO classification of Tumours of the urinary system and male genital organs. International Agency for Research on Cancer.

[6] Makise N, Morikawa T, Kawai T, Nakagawa T, Kume H, Homma Y (2015). Squamous differentiation and prognosis in upper urinary tract urothelial carcinoma. Int J Clin Exp Pathol.

[7] Terauchi T, Nakada S, Takenaka M, Mizuguchi S, Okanemasa Y, Tsubata Y (2020). Intracytoplasmic lumen in urine cytology predicts worse prognosis in non-muscle-invasive bladder cancers. Acta Cytol.

[8] Kuromoto A, Namiki S, Satake Y, Yamashita S, Mitsutzuka K, Saito H, Kaiho Y, Arai Y (2014). Upper tract urothelial carcinoma associated with Giant Hydronephrosis due to ligation of a ureter: a case report. Hinyokika Kiyo (Japanese).

